# Comparing Minimally Invasive Percutaneous Plate Osteosynthesis With Interlocking Intramedullary Nail Fixation for the Management of Adult Extra-Articular Distal Tibial Fractures: A Comprehensive Systematic Review and Meta-Analysis

**DOI:** 10.7759/cureus.49214

**Published:** 2023-11-21

**Authors:** Ahmed Elnewishy, Mohamed Elkholy, Ahmed Hamada, Mohamed Salem

**Affiliations:** 1 Trauma and Orthopedics, Kasr Al-Ainy Medical School, Cairo, EGY; 2 Orthopedic Surgery, Al Helal Hospital, Cairo, EGY; 3 Trauma and Orthopedics, King's College Hospital, London, GBR; 4 General Surgery, King's Mill Hospital, Nottingham, GBR

**Keywords:** interlocking intramedullary nail fixation, distal tibial fractures, plate osteosynthesis, extra-articular distal tibial fractures, invasive percutaneous plate osteosynthesis

## Abstract

Intramedullary nailing (IMN) and minimally invasive percutaneous plate osteosynthesis (MIPPO) fixation are both viable approaches for managing distal tibia fractures. IM nailing offers advantages in terms of shorter operation time, faster union, and reduced infection rates, yet it may lead to alignment issues and residual knee pain. Conversely, MIPPO fixation provides better alignment and minimizes knee discomfort but comes with a higher risk of soft-tissue complications and hardware irritation. Notably, this review reveals that MIPPO is associated with a greater risk of both superficial (15% vs. 7% for IMN) and deep infections (14% vs. 6.3% for IMN). This study aims to comprehensively assess the optimal surgical approaches for distal tibia fractures by comparing clinical and functional outcomes between MIPPO and interlocking IMN techniques in treating extra-articular distal tibial fractures. Key outcome parameters include operation duration, union time, non-union occurrence, malunion cases, infection rates, secondary surgical interventions, and functional results, as indicated by quality of life and ankle scores. Regarding union complications, it is notable that IMN demonstrates a higher incidence of malunion, affecting 14.7% of patients compared to 8.8% in the MIPPO fixation group. Interestingly, both treatment methods exhibit a similar incidence of non-union, occurring in 3.5% of patients in both groups. Furthermore, when assessing the union time, IMN fixation notably achieves significantly shorter union times, especially evident in AO 43A fracture types and closed fractures. The mean time for union is 18 weeks with IMN compared to 20 weeks with MIPPO fixation. In our analysis of nine studies involving 813 patients, the reported operation times revealed an overall weighted mean operation time of 74.1 minutes (ranging from 56.4 to 124 minutes) for IMN and 85.4 minutes (ranging from 51.4 to 124 minutes) for MIPPO fixation. Notably, the operation time for IMN was significantly shorter compared to MIPPO, showing a weighted mean difference (WMD) of -11.24 minutes, with a 95% confidence interval (CI) ranging from -15.44 to -7.05 (P<0.05). This difference exhibited significant moderate heterogeneity (I^_2_^ = 68%). In light of this comprehensive study, both MIPPO and IMN emerge as equally effective therapeutic options for addressing functional outcomes in distal tibial extra-articular fractures. While IMN offers several advantages, including lower infection rates, reduced implant irritation, shorter operation time, and earlier weight-bearing and union, it is associated with a heightened risk of malunion and anterior knee pain. Consequently, the choice of implant should be tailored on a case-by-case basis. Patients at elevated infection risk, stemming from factors, such as advanced age, comorbidities, smoking, or severe soft tissue injuries, are better suited for nail treatment. Conversely, MIPPO fixation may present a more advantageous choice for young, active, and healthy patients, given its ability to mitigate the risk of knee pain and malunion.

## Introduction and background

To date, there is no consensus on the best mini-invasive internal fixation method for extra-articular distal tibial fractures. This may be attributed to many reasons. Basically, there is a controversy over the use of the term "distal tibial fractures." Some authors use the term to describe the distal metaphyseal fractures as defined by one muller square as Giannoudis et al. [1]. Others use distal tibial fractures to refer to distal shaft fractures (metadiaphyseal region) from 4 cm to 11 cm from the plafond, as Polat et al. [2]. Others use the term for both regions, describing them as "2 muller squares," as Mauffrey et al. [3].

Surprisingly, there is also a controversy over using the terms “extra-articular” or “non- articular” fractures. Most authors consider the absence of an articular extension to be mandatory to use the term, while others, as Casstevens et al. [4] and Bedi et al. [5], define non-articular or extra-articular fractures as “those with no or with a simple extension of a non- displaced fracture line into the plafond.”

Moreover, fractures in the distal tibial region are still not considered a separate entity and clearly distinguished from pilon fractures or tibial shaft fractures, despite the vast difference in the anatomy between these regions. This is evident in orthopaedic trauma textbooks [6,7] and the available AO classification.

The treatment principles for distal tibial fractures require distinct consideration compared to proximal diaphyseal fractures and distal intra-articular pilon fractures. In addition, the management of associated fibular fractures lacks consensus, with differing opinions on its necessity for preventing malalignment or its potential association with non-union.

Dealing with distal tibial fractures in skeletally mature patients without articular involvement poses challenges due to unique anatomical characteristics, such as proximity to the skin, limited blood supply, and adjacency to the ankle joint, providing only a short segment for implant fixation.

Traditionally, surgical management involved open reduction and internal fixation, but advancements in implant designs have led to alternative strategies, like minimally invasive percutaneous plate osteosynthesis (MIPPO) and intramedullary nailing (IMN) gaining popularity for addressing these fractures.

External fixation, combined with limited open reduction and internal fixation (EF + LORIF), is advocated for fractures with minimal soft tissue complications, demonstrating favorable outcomes and minimizing local soft tissue irritation. External fixation serves a crucial role in temporarily stabilizing open fractures with extensive soft tissue injuries and definitively managing extremely distal fractures where secure fixation with a nail or plate is challenging.

While these techniques are successful in preserving reduction and promoting stable union, it is imperative to acknowledge their unique advantages and disadvantages. No fixation technique universally suits all combinations of bony and soft tissue injuries in distal tibial fractures. Therefore, a thorough evaluation of each technique's strengths, weaknesses, and appropriate applications is essential for informed surgical decision-making.

Aim of the study

This study aims to comprehensively review the optimal surgical approaches for distal tibia fractures by assessing and contrasting the clinical and functional outcomes between MIPPO and IMN techniques in the management of extra-articular distal tibial fractures. Key outcome measures encompass operation duration, time to achieve union, non-union incidence, malunion occurrences, infection rates, secondary surgical interventions, and functional outcomes, which include assessments of quality of life scores and ankle function scores.

## Review

Patients and methods

Objective

The overarching goal of this study is to conduct a thorough and insightful evaluation of the optimal surgical interventions for distal tibia fractures. This entails a comprehensive comparative analysis of the clinical and functional outcomes associated with two primary surgical techniques, namely, MIPPO and IMN, in the management of extra-articular distal tibial fractures.

Methodology

The research methodology adhered rigorously to the standardized procedures outlined in the Cochrane Handbook, ensuring a robust and systematic approach. The reporting of findings was conducted in accordance with the guidelines set forth by the Preferred Reporting Items for Systematic Reviews and Meta-analyses (PRISMA) statement [8].

Criteria for Study Inclusion

Types of studies: The study exclusively focused on randomized controlled trials (RCTs), providing a methodologically robust foundation for the comparative assessment of postoperative outcomes between MIPPO and ILN in patients with extra-articular distal tibial fractures.

Types of participants: The inclusion criteria targeted skeletally mature adult humans diagnosed with specific types of distal tibial fractures, namely, AO 42 type (A, B, C) or AO 43A type (A1, A2, A3). Importantly, these participants had no articular involvement or severe soft tissue injury.

Types of interventions: The investigation specifically delved into the comparison between MIPPO and interlocking IMN for the fixation of extra-articular distal tibial fractures. The minimum follow-up duration was set at six months to capture longer-term outcomes.

Types of outcome measures: The comprehensive array of outcome measures encompassed various facets crucial to the assessment of surgical efficacy. These included but were not limited to operation time, time to union, full weight-bearing time, weight-bearing time, radiation time, infection rates (superficial and deep), union complications (non-union and malunion), secondary operations, anterior knee pain, and a spectrum of functional scores, such as Disability Rating Index (DRI) [9], Foot Function Index (FFI) [10], American Orthopaedic Foot and Ankle Society (AOFAS) score [11], and Olerud-Molander Ankle Score (OMAS) [12].

Inclusion Criteria

The study considered English literature exclusively, with a focus on RCTs published between 2000 and January 1, 2021. Specifically, the studies described the comparative analysis of MIPPO versus interlocking IMN in the fixation of extra-articular distal tibial fractures in adult human subjects.

Exclusion Criteria

Rigorous exclusion criteria were applied to maintain the precision and relevance of the study. Articles involving intra-articular distal tibial fractures, cadaver studies, animal studies, non-English studies, and those falling outside the category of RCTs (including case reports, case series studies, cross-sectional studies, cohort studies, and clinical trials) were excluded.

Search Strategy

The study employed a meticulous systematic literature search strategy. This involved a comprehensive exploration of the Cochrane library and PubMed databases, covering the expansive time frame from 2000 to January 1, 2023. The search was executed using a strategically crafted set of keywords, including "extra-articular distal tibial fractures," "distal tibial fractures," "interlocking intramedullary nail," "minimally invasive plate/plates," "MIPPO," "percutaneous plate/plates," "minimally invasive percutaneous plate/plates," and "locking plate."

Methods of the review

The methodology employed for this review is detailed as follows:

Locating and Selecting Studies

Titles and abstracts of articles identified through the search strategy were thoroughly reviewed. Articles that appeared to meet the inclusion criteria were retrieved in full for detailed examination.

Data Extraction

A structured approach was applied during the data extraction process, with information from each included study organized into spreadsheets. Details extracted included authors' names, study year, publication year, study location (country), study design, study duration, journal of publication, study subjects, and patient demographics. Patient-related factors and fracture characteristics, such as gender, age, AO/OTA classification, and Gustilo classification for open/closed fractures, were systematically collected. Outcome measures relevant to the study's objectives were documented for comprehensive analysis.

Statistical Analysis

Data analysis was conducted using Review Manager 5.4 software. Dichotomous variables were expressed as risk ratios (RRs) with 95% confidence intervals (CIs), while continuous data were presented as mean differences (MDs) or standardized mean differences (SMDs). Heterogeneity was assessed using the I^2^ statistic, and Cochrane risk-of-bias criteria were applied for methodological quality assessment. Sensitivity and subgroup analyses were performed as needed, and forest plots were utilized to visually represent results. A significance level of P<0.05 was considered statistically significant.

Study Selection Process

**Figure 1:**
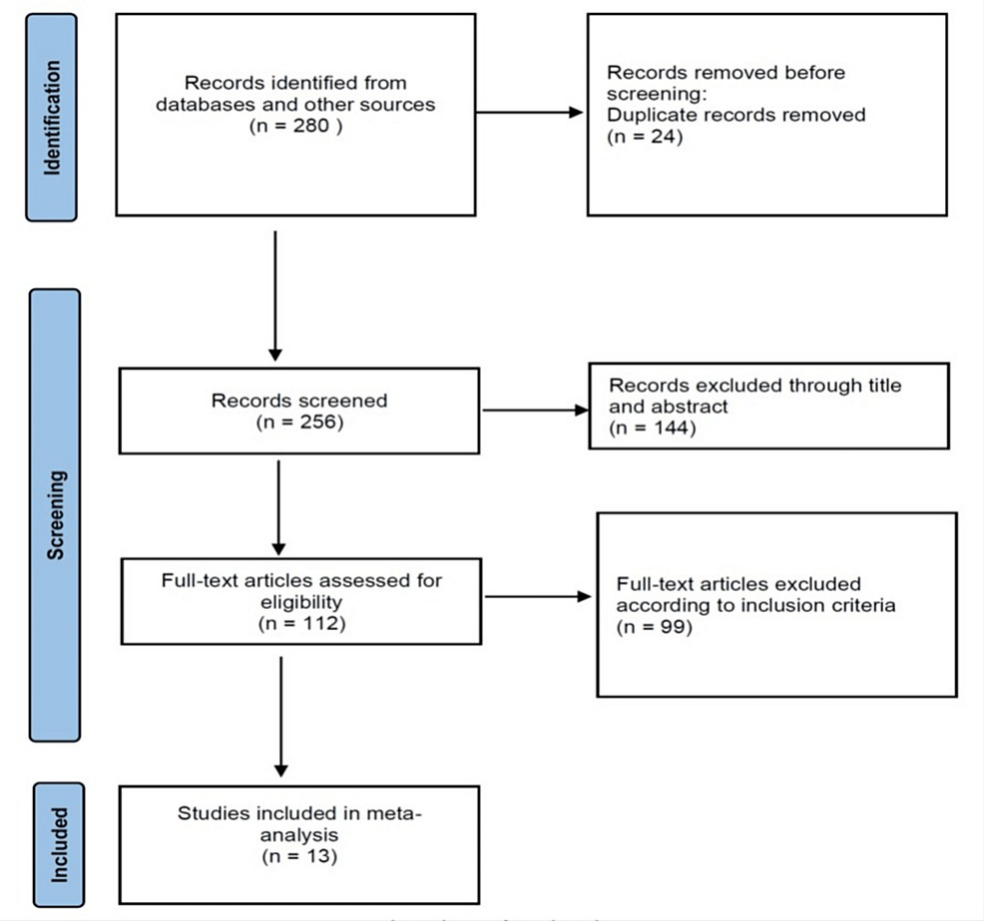
Flow chart of the study selection process.

The initial search identified 256 items, excluding duplicates. After screening titles and abstracts, 144 studies were excluded. Following a thorough full-text screening, an additional 98 studies that did not meet eligibility criteria were excluded. Ultimately, 13 RCTs involving 1,017 patients were deemed eligible for data extraction and subsequent meta-analysis. The entire study selection process is visually presented in a flow diagram for reference (Figure 1).

Risk assessment and quality evaluation of studies

The assessment of potential bias and the quality of the included trials was conducted using the Cochrane Collaboration’s risk of bias tool, encompassing seven domains [13]. Trials were meticulously graded as having a low, high, or unclear risk of bias based on specific criteria for each domain:

Allocation

Randomization processes varied among studies. Two studies [14,15] utilized computer-assisted tools, implementing allocation concealment through opaque envelopes. Additional studies, including Mauffrey et al. [3], Daolagupu et al. [16], Prasad et al. [16], and Costa et al. [17], employed computer random number generators. Notably, Costa et al. [17] utilized a secure, centralized, web-based randomization service with a minimization algorithm. Polat et al. [2] and Kariya et al. [18] employed coin flipping for randomization without explicitly reporting allocation concealment. Ali et al. [19] randomized patients using a random number table, while Lakhotia et al. [20] and Wani et al. [21] employed permuted block randomization without detailing allocation concealment. Two studies [22,23] did not provide a comprehensive method of randomization and allocation concealment.

Blinding

**Figure 2:**
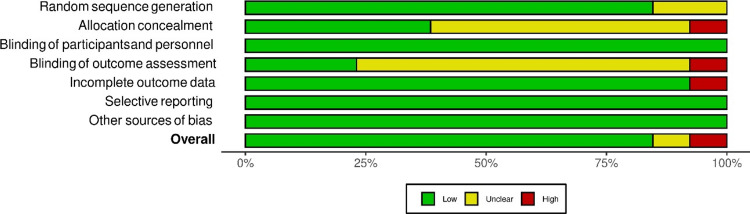
Risk-of-bias assessment of the included studies and summary plot

None of the studies reported specifics about blinding participants and personnel. Given the inherent differences in surgical incisions for nailing and plating, blinding of surgeons and participants was deemed impractical. Consequently, a judgment of low risk of performance bias was applied, as the outcomes were not likely to be influenced by the lack of blinding. Guo et al. [22] described outcome assessment by doctors involved in the treatment, leading to a considered high risk of detection bias. By contrast, Costa et al. [17], Mauffrey et al. [3], and Fang et al. [14] underwent a review by an independent assessor not involved in other data collections or analyses, resulting in a low risk of detection bias (Figure 2).

Incomplete Outcome Data

The risk of bias for incomplete outcome data is considered a less than 80% completion rate in the treatment group as indicative of a high risk. Consequently, Costa et al.'s study [17] was deemed to have a high risk of attrition bias due to a proportion of missing outcomes sufficient to potentially induce clinically relevant bias in the intervention effect estimate.

Selective Reporting

Although trial registration documents or protocols were unavailable for all studies, it is evident that all pre-specified and expected outcomes of interest were reported, ensuring transparency and completeness in reporting.

In this meta-analysis, a total of 13 RCTs involving 1,017 patients were included. Below is a summary table highlighting the key characteristics of these included studies (Table 1).

**Table 1:** Characteristics of included studies. MIPPO: minimally invasive percutaneous plate osteosynthesis; IMN: intramedullary nailing

Author	Year	Country	Study period	Total number	MIPPO	IMN	Follow-up (months)	Journal
Ali et al. [19]	2017	India	2013-2017	60	30	30	>6	Trauma Monthly Journal
Costa et al. [17]	2018	UK	2013-2016	321	160	161	>6	Health Technology Assessment
Daolagupu et al. [16]	2017	India	2014-2015	42	21	21	>6	Indian Journal of Orthopaedics
Fang et al. [14]	2016	China	2005-2013	56	28	28	28.9 (10)	Orthopedics Journal
Guo et al. [22]	2010	China	2005-2008	85	41	44	>6	The Journal of Bone and Joint Surgery
Kariya et al. [18]	2020	India	2015-2018	142	69	73	>6	European Journal of Orthopaedic Surgery & Traumatology
Lakhotia et al. [20]	2020	India	2016-2017	50	25	25	>6	International Journal of Research in Orthopaedics
Li et al. [15]	2014	Japan	2002-2012	92	46	46	14.9 (1.1)	International Orthopaedics (SICOT)
Mauffrey et al. [3]	2012	USA	2008-2009	24	12	12	>6	The Journal of Bone and Joint Surgery
Pawar et al. [23]	2014	India	NR	30	15	15	>6	Journal of Evolution of Medical and Dental Sciences
Polat et al. [2]	2015	Turkey	2009-2012	25	15	10	23.1 (9.4)	The Japanese Orthopaedic Association
Prasad et al. [13]	2017	Multi-center	2012-2016	30	15	15	>6	International Journal of Orthopaedics Sciences
Wani et al. [21]	2017	India	2014-2016	60	30	30	11.5 (4.5)	Ortopedia Traumatologia Rehabilitacja

Patient characteristics

Age

**Figure 3:**
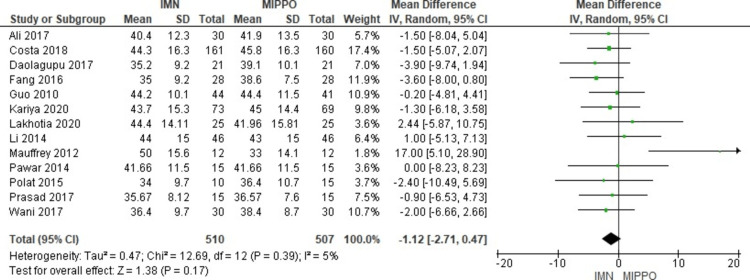
Age forest plot for the included studies

All studies reported the age of participants: 510 (50.14%) patients were treated with IMN with a weighted mean age of 40.3 years (range 34-50 years), and 507 patients (49.86%) were treated with MIPPO fixation with a weighted mean age of 41.5 years (range 33-45.8 years) (Figure 3).

Sex

**Figure 4:**
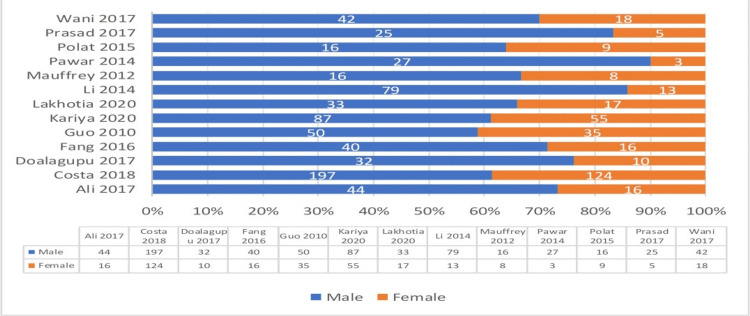
Sex distribution in the included studies

All studies reported the gender of participants: 510 patients were treated with IMN, with 347 (68%) being males and 163 (32%) females, and 507 patients were treated with MIPPO fixation, with 341 (67%) being males and 166 (33%) females. The range of male patients in the IMN group was 7-96, and the MIPPO fixation group was 7-101. Meanwhile, the range of female patients in the IMN group was 1-65, and the MIPPO fixation group was 2-59 (Figure 4).

Diabetes

Two studies reported on diabetes: Fang et al. [14] reported one patient in the MIPPO group, and Costa et al. [17] reported seven patients in the MIPPO group and another six patients in the IMN group.

**Figure 5:**
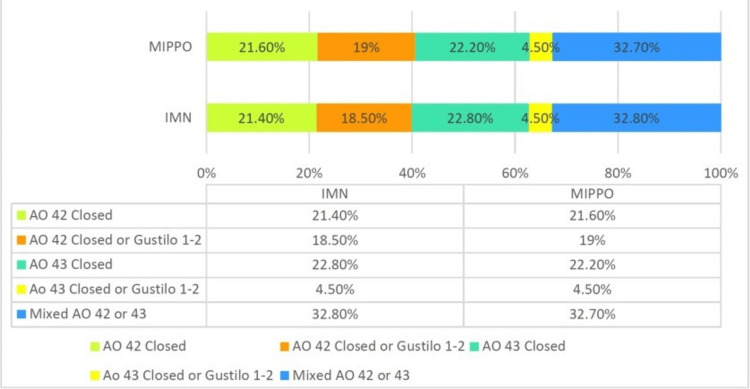
Patient percentage according to injury characteristics Source: Kitaoka et al. [11]

Injury characteristics: A total of 13 studies were analyzed; nine studies focused on closed patients, two studies focused on closed or Gustilo 1, and two studies focused on closed or Gustilo 1-3. The fracture pattern in all studies was classified according to the AO/OTA classification: four studies focused on (43 A1, A2, A3), three studies focused on (42 A1, A2, A3), two studies focused on (42 A, B, C), three studies focused on (42A, B, C, 43A), and one study focused only on (42A2) (Figure 5, Table 2).

**Table 2:** Injury characteristics of the included studies IMN: intramedullary nailing; MIPPO:minimally invasive percutaneous plate osteosynthesis

Author	Wound type	Open fractures		Closed fractures	
		IMN	MIPPO	IMN	MIPPO
Ali et al. (75)	Closed or open G1	10	7	20	23
Costa et al. (74)	Closed	NR*	NR*	161	160
Daolagupu et al. (72)	Closed	NR	NR	21	21
Fang et al. (70)	Closed or open G1-2	4	6	24	22
Guo et al. (20)	Closed	NR	NR	44	41
Kariya et al. (60)	Closed	NR	NR	73	69
Lakhotia et al. (76)	Closed	NR	NR	25	25
Li et al. (71)	Closed or open G1-2	17	14	29	32
Mauffrey et al. (3)	Closed or open G1	NR	NR	NR	NR
Pawar et al. (78)	Closed	NR	NR	15	15
Polat et al. (2)	Closed	NR	NR	10	15
Prasad et al. (73)	Closed	NR	NR	15	15
Wani et al. (77)	Closed	NR	NR	30	30

Intervention Characteristics

All studies used minimally invasive locking plate (MIPPO) fixation and reamed interlocking nail fixation, except Fang et al. [14] who used unreamed tibial nails. In all the studies, the plate was positioned medially on the distal tibia. In the IMN group, the patella tendon splitting incision was used in seven studies [2,18,14,15,19,20,23]. In one study, the patella tendon was retracted laterally [3], and five studies did not specify the used incision [12,3,22,17,21]. All studies used standard interlocking nails, except Ali et al. [19] who used expert interlocking nails. Costa et al. [17] and Mauffrey et al. [3] used Poller screws. A postoperative short-leg splint or cast was used for three weeks in one study [22]. Eight studies allowed an active range of motion in the ankle and knee joints during the first postoperative week [3,14-17,20,23], while the remaining did not specify the immobilization protocol [2,18,19,21]. In all the studies, the allowance of weight bearing was assessed individually, usually only after a bony callus was seen in X-ray images.

Results

Outcome Measures

**Figure 6:**
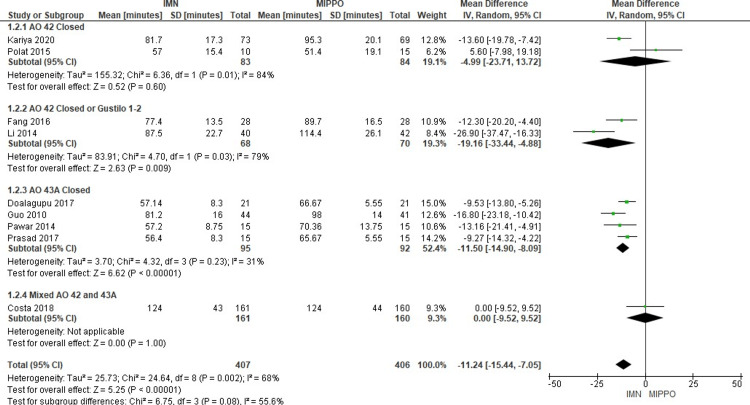
Forest plot for operation time in minutes

Regarding the operation time, data from nine studies involving 813 patients were analyzed (Figure 6). The overall weighted mean operation time for IMN procedures was 74.1 minutes, ranging from 56.4 to 124 minutes, while MIPPO procedures had an overall weighted mean operation time of 85.4 minutes, ranging from 51.4 to 124 minutes. Notably, IMN surgeries exhibited a significantly shorter operation time compared to MIPPO, with a weighted mean difference (WMD) of -11.24 minutes and a 95% CI ranging from -15.44 to -7.05 (P<0.05). Heterogeneity was observed, with a significant moderate heterogeneity score (I^2^ = 68%).

The subgroup analysis revealed significant differences in the AO 42 closed or Gustilo 1-2 and AO 43A closed subgroups, but not in the AO 42 closed subgroup.

Time to Union (weeks)

**Figure 7:**
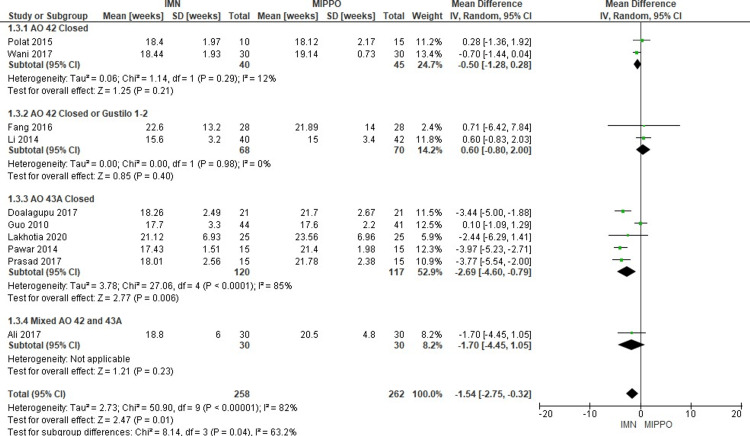
Forest plot for time to union in weeks

**Figure 8:**
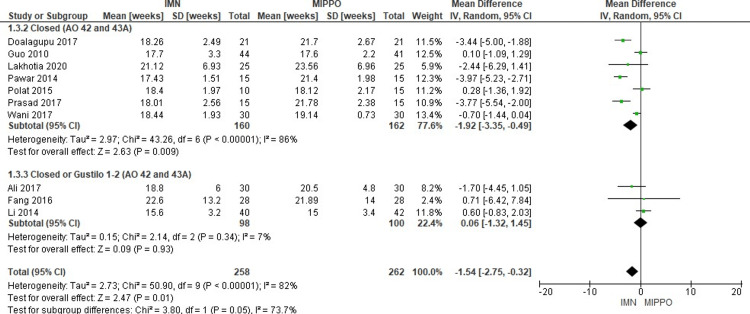
Closed and open subgroups forest plot for time to union in weeks

Ten studies including 520 patients reported on the time to union, with an overall weighted mean time to union of 18 weeks (range 15.6-22.6 weeks) in IMN and 20 weeks (range 15-25.7 weeks) in MIPPO (Figure 7). The time to union in IMN was significantly shorter compared to that in MIPPO (WMD -1.54 weeks, 95% CI -2.75 to -0.32, P = 0.01) with significant severe heterogeneity (I^2^ = 82%) (Figure 8).

Subgroup analysis: Only the AO 43A closed subgroup showed a significantly shorter time to union in IMN compared to the MIPPO, but there was no statistically significant difference in the rest of the subgroups.

Radiation Time (Minutes)

**Figure 9:**
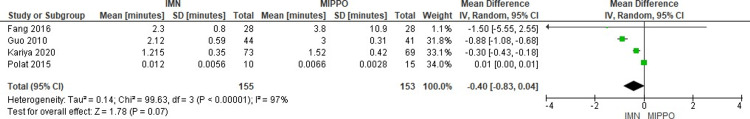
Forest plot for radiation time

Four studies including 308 patients reported on radiation time with an overall weighted mean radiation time of 1.1 minutes (range 0.01-2.3 minutes) in IMN and 1.5 minutes (range 0.006-3.8 minutes) in MIPPO (Figure 9). There was no significant difference in radiation time between both methods (WMD -0.4 minutes, 95% CI -0.83 to 0.04, P = 0.07) with significant severe heterogeneity (I^2^ = 97%).

**Figure 10:**
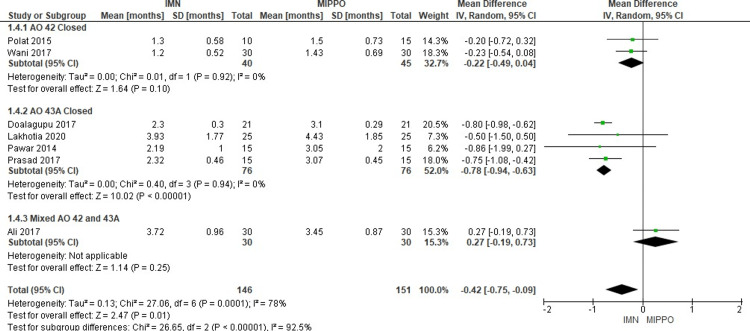
Forest plot for time to full weight-bearing

In the evaluation of time to full weight-bearing across seven studies involving 297 patients, it was found that patients treated with an IMN achieved full weight-bearing at a weighted mean time of 2.2 months, ranging from 1.2 to 3.9 months (Figure 10). By contrast, patients treated with MIPPO fixation reached full weight-bearing at a weighted mean time of 2.7 months, ranging from 1.4 to 4.4 months. Significantly, the time to weight-bearing was notably shorter for patients undergoing IMN treatment, with a WMD of -0.42 months and a 95% CI ranging from -0.75 to -0.09 (P = 0.01). Substantial heterogeneity was observed, with an I^2^ value of 78%.

The subgroup analysis revealed a statistically significant difference in the time to full weight-bearing between the IMN and MIPPO groups only within the AO 43A closed subgroup. In this specific subgroup, patients treated with IMN achieved full weight-bearing significantly sooner than those in the MIPPO group. However, in the remaining subgroups, there were no statistically significant differences observed in the time to full weight-bearing between the two treatment modalities.

Complications

**Figure 11:**
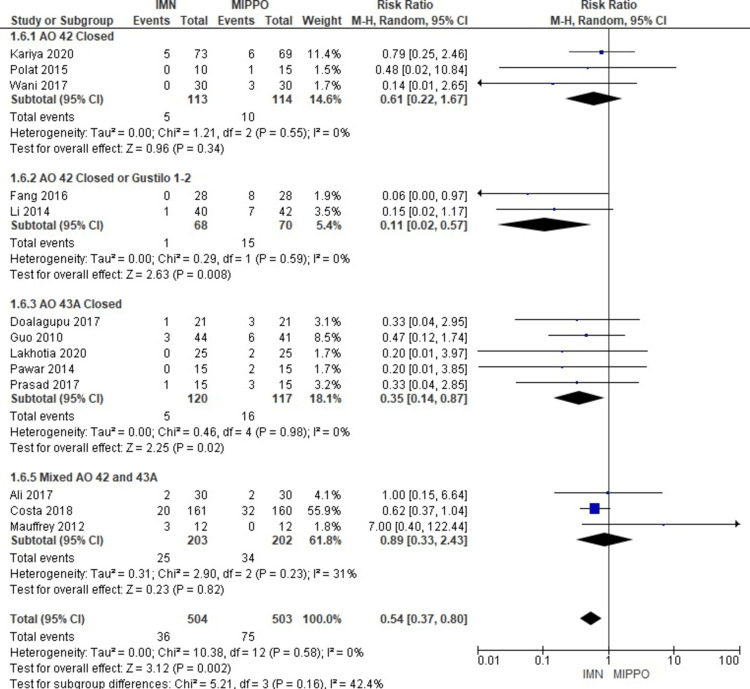
Forest plot for superficial infection

**Figure 12:**
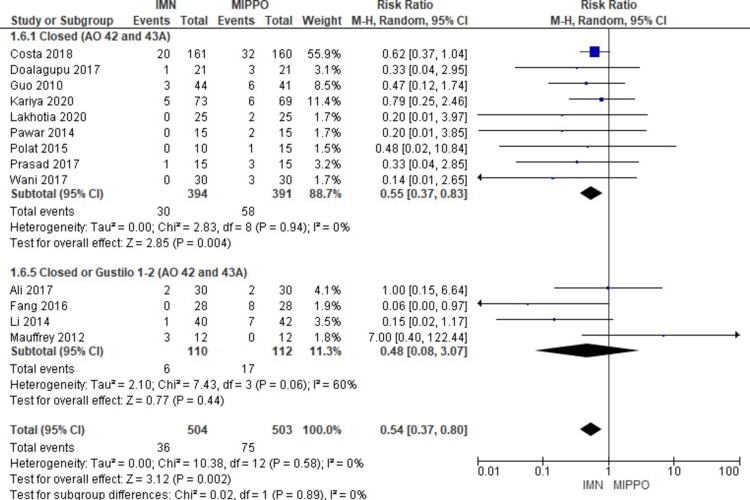
Closed and open subgroups forest plot for superficial infection

The analysis of superficial infection, encompassing all studies involving 1,007 patients, revealed a statistically significant difference (Figure 11). Patients treated with MIPPO had a substantially higher risk of superficial infection compared to those treated with IMN, as indicated by a weighted RR of 0.54 and a 95% CI ranging from 0.37 to 0.8 (P = 0.002) (Figure 12). Importantly, there was no heterogeneity observed in this analysis (I^2^ = 0%). Specifically, superficial infection occurred in 7% of patients treated with IMN and 15% of patients treated with MIPPO, reflecting a weighted risk difference (RD) of -8%.

The subgroup analysis unveiled that patients treated with MIPPO had a significantly higher risk of superficial infection in specific subgroups, including AO 42 closed fractures and Gustilo 1-2 wounds, as opposed to no statistically significant difference in the AO 42 closed and mixed AO 42 and AO 43A subgroups. When combining the data from all closed fractures, whether AO 42 or AO 43A, there was a significant difference favoring IMN regarding superficial infection. However, in the pooled analysis of all closed or open fractures, encompassing AO 42 or AO 43A classifications, there was no statistically significant difference observed between the two treatment methods in terms of superficial infection rates.

**Figure 13:**
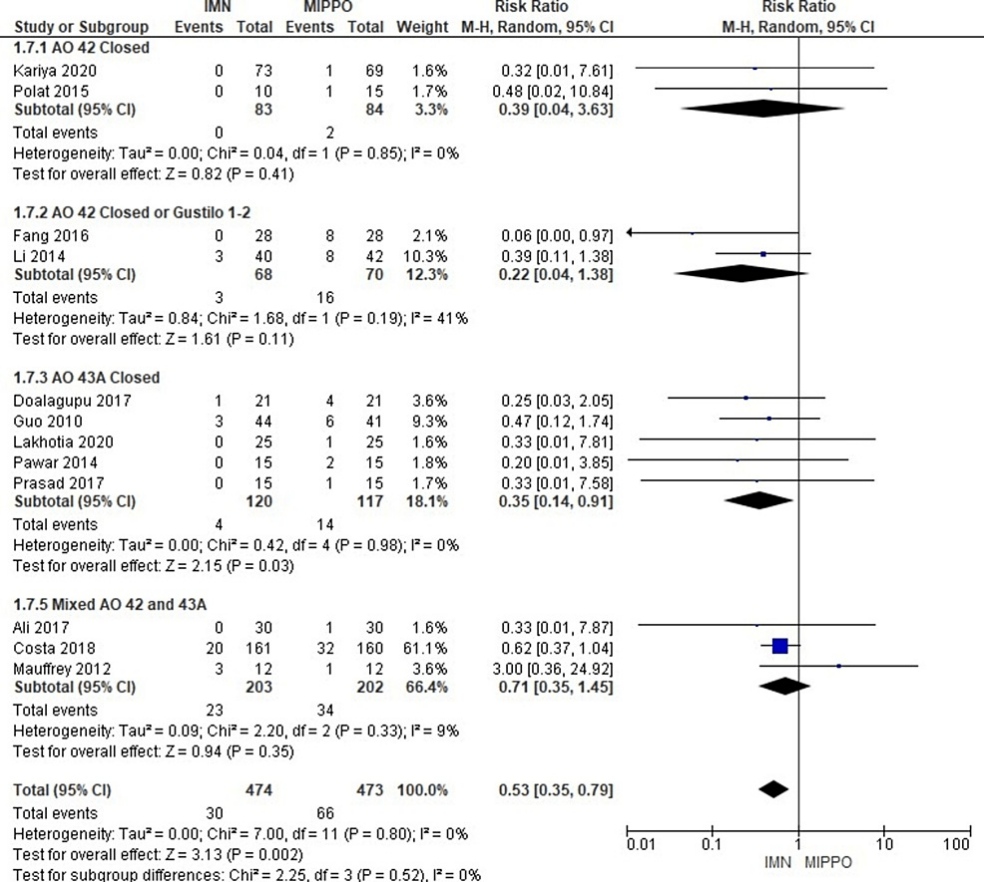
Forest plot for deep infection

**Figure 14:**
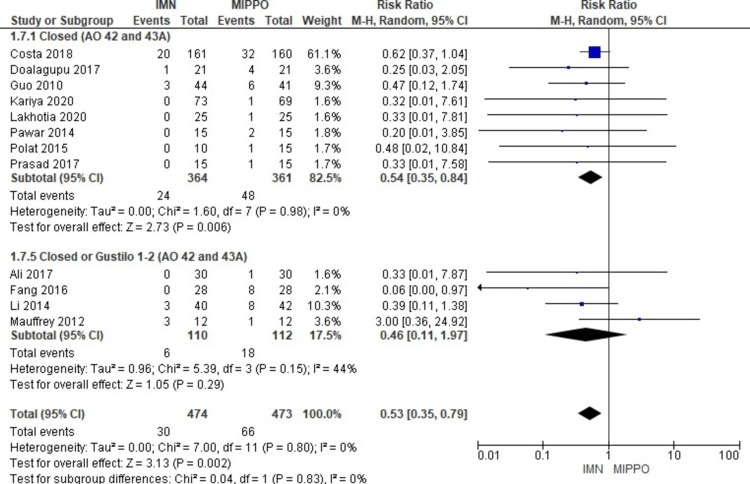
Closed and open subgroups forest plot for deep infection

The analysis of deep infection, involving 12 studies comprising 947 patients, revealed a statistically significant difference (Figure 13). Patients who underwent MIPPO fixation had a significantly higher risk of deep infection compared to those treated with IMN, as evidenced by a weighted RR of 0.53 and a 95% CI ranging from 0.35 to 0.79 (P = 0.002) (Figure 14). Importantly, there was no observed heterogeneity in this analysis (I^2^ = 0%). Specifically, deep infection occurred in 6.3% of patients treated with IMN and 14% of patients after MIPPO fixation, reflecting a weighted RD of -7%.

The subgroup analysis revealed that only the AO 43A closed subgroup showed a significantly higher risk for deep infection among patients treated with MIPPO fixation, but there was no statistically significant difference in the rest of the other subgroups. For pooled analysis of subgroups, all closed fractures whether AO 42 or AO 43A showed a significant difference favouring IMN. The pooled data of all closed or open AO 42 or AO 43A closed showed no statistically significant difference between IMN and MIPPO groups.

**Figure 15:**
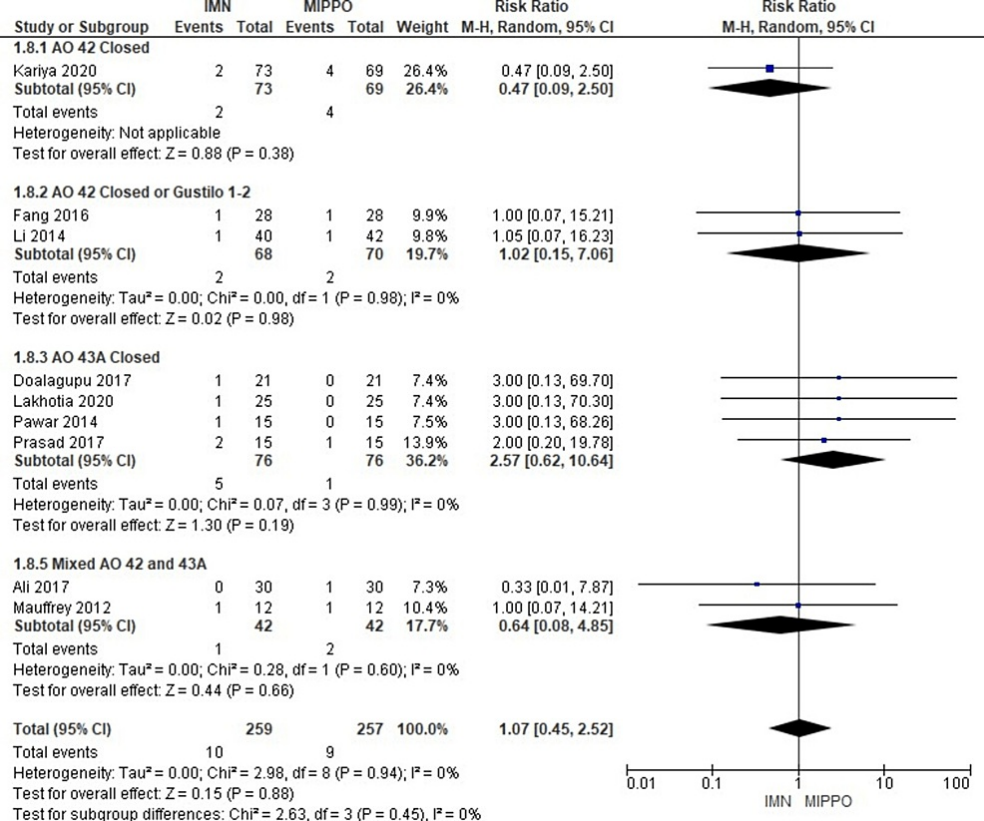
Forest plot for non-union

**Figure 16:**
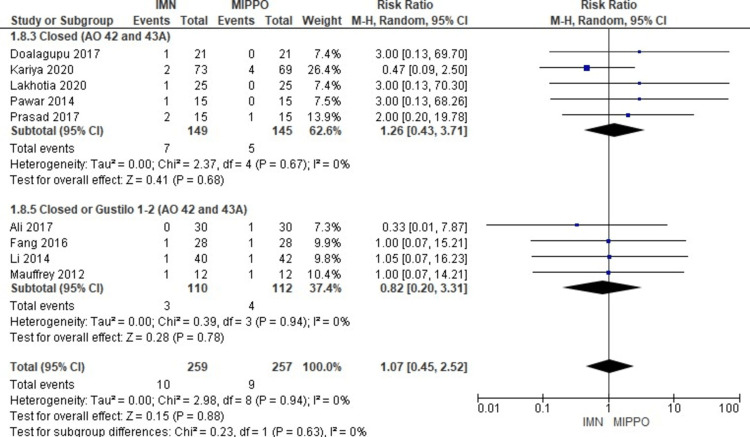
Closed and open subgroups forest plot for non-union

The analysis of non-union, encompassing data from nine studies with a total of 516 patients, revealed no significant difference in the risk for non-union between the two treatment groups (Figure 15). The weighted RR was 1.07, with a 95% CI ranging from 0.45 to 2.52 (P = 0.88), and there was no heterogeneity observed (I^2^ = 0%) (Figure 16). Non-union occurred in 3.5% of patients in both treatment groups, resulting in a weighted RD of 0%.

The subgroup analysis revealed that there was no significant difference in risk for non-union between IMN and MIPPO fixation in all subgroups.

**Figure 17:**
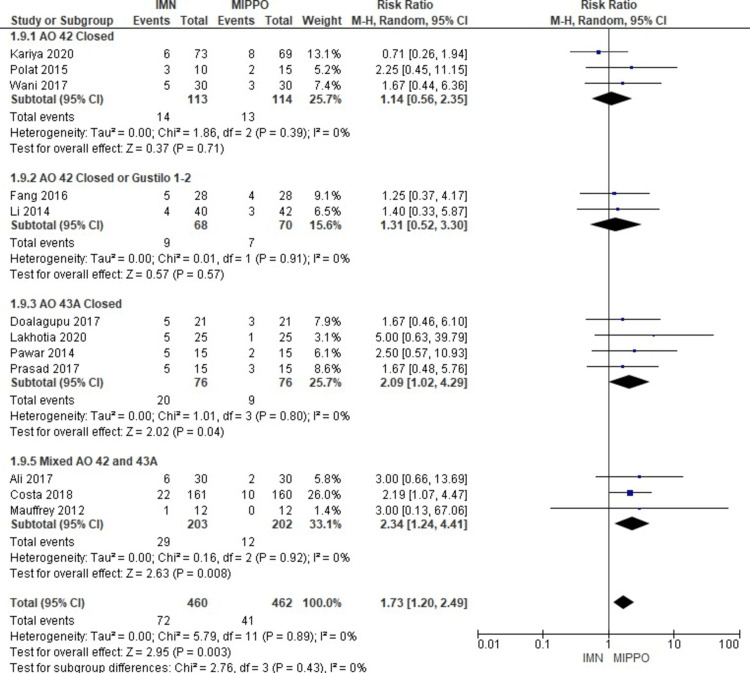
Forest plot for malunion

**Figure 18:**
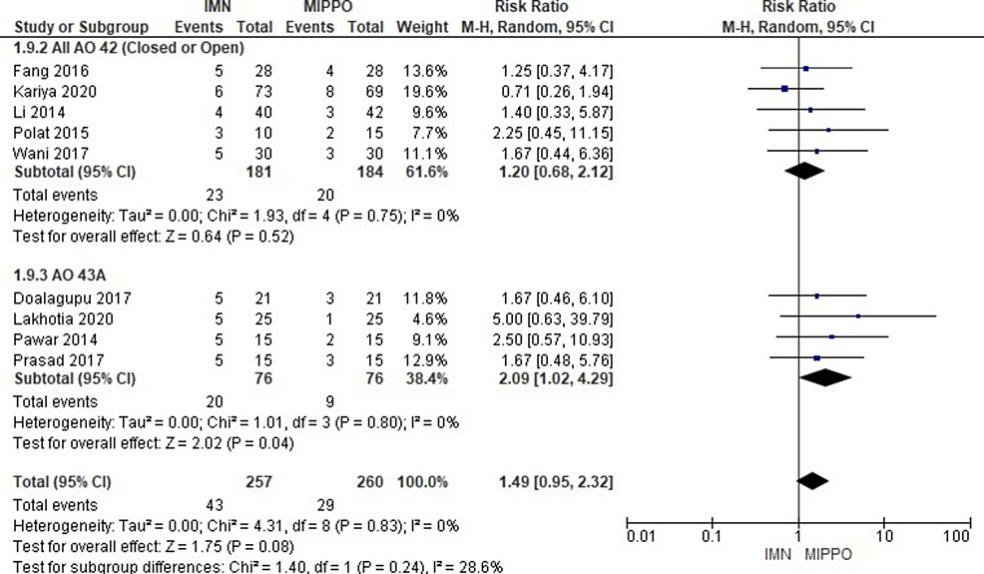
All AO 42 and AO 43A subgroups' forest plot for malunion

In the assessment of malunion across 12 studies involving 922 patients, a significant difference was observed (Figure 17). Patients who underwent MIPPO fixation had a significantly lower risk of malunion compared to those treated with IMN. This difference was quantified by a weighted RR of 1.66 and a 95% CI ranging from 1.15 to 2.39 (P = 0.007) (Figure 18). Notably, there was no observed heterogeneity (I^2^ = 0%) in this analysis. Specifically, malunion occurred in 14.7% of patients treated with IMN and 8.8% of patients after MIPPO fixation, resulting in a weighted RD of 5%.

The subgroup analysis revealed that the malunion rate in the IMN was significantly higher compared to the MIPPO in the mixed (AO 42 and AO 43A) and AO 43A subgroups. There was no statistically significant difference in the rest of the other subgroups.

**Figure 19:**
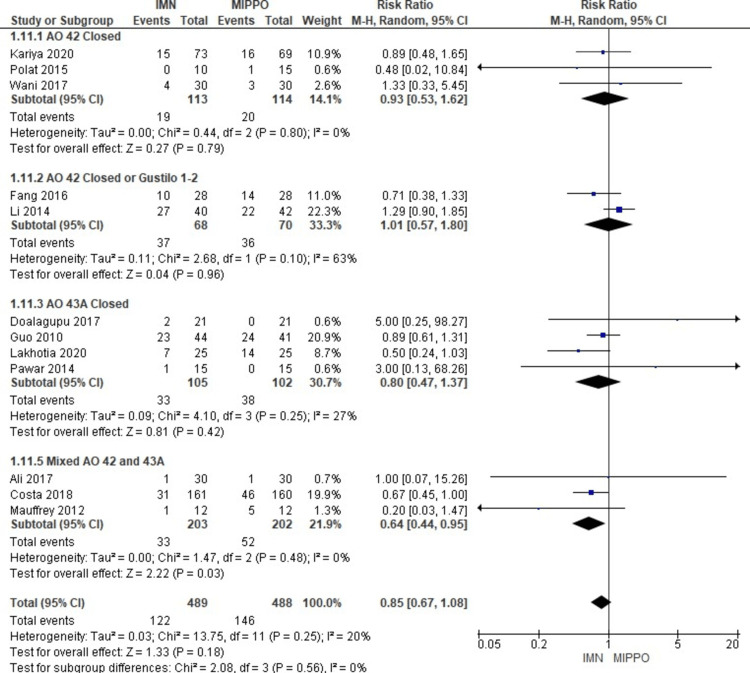
Forest plot for secondary operations

**Figure 20:**
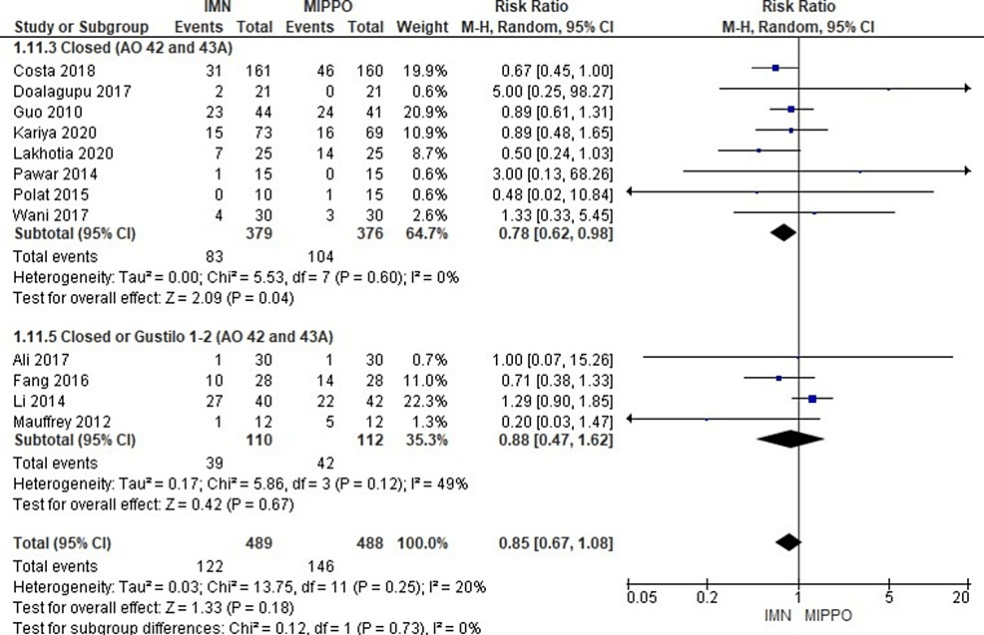
Closed and open subgroups forest plot for secondary operations

In the evaluation of secondary operations across 12 studies involving 977 patients, no significant difference was observed in the risk of requiring secondary operations between IMN and MIPPO (Figure 19). The weighted RR was 0.85, with a 95% CI ranging from 0.67 to 1.08 (P = 0.18). Mild heterogeneity was noted in this analysis (I^2^ = 20%) (Figure 20). Secondary operations were necessary in 25% of patients in the IMN fixation group and 30% of patients in the MIPPO group, resulting in a weighted RD of 3%.

The subgroup analysis revealed that there was a significantly higher risk for secondary operations among patients treated with MIPPO in the mixed (AO 42 and 43A) and closed (AO 42 and 43A). There was no statistically significant difference in the rest of the other subgroups.

**Figure 21:**
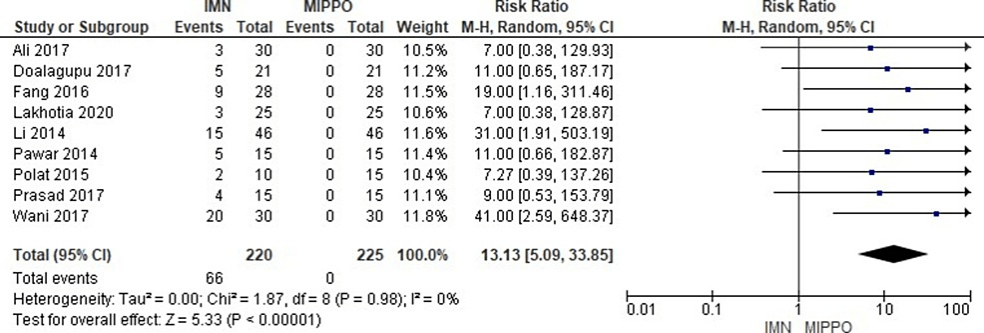
Forest plot for anterior knee pain

The analysis of anterior knee pain, which included data from 10 studies involving 445 patients, revealed a significant difference (Figure 21). Patients who underwent IMN treatment had a significantly higher risk of experiencing anterior knee pain compared to those treated with MIPPO. The weighted RR was notably high at 13.1, with a 95% CI ranging from 5 to 33.8 (P < 0.05). Importantly, there was no observed heterogeneity (I^2^ = 0%) in this analysis. Specifically, anterior knee pain was reported in 28% of patients treated with IMN, while it was absent (0%) after MIPPO fixation, resulting in a weighted RD of 28%.

Functional Outcomes

**Figure 22:**
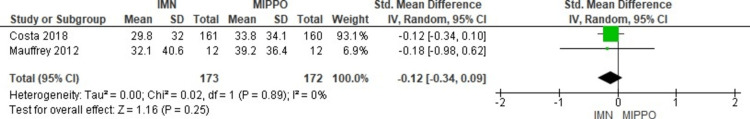
Forest plot for quality-of-life score (Disability Rating Index)

In the assessment of the general quality of life scores at six to 12 months postoperatively, data from only two studies involving 345 patients were available (Figure 22). Both studies utilized the Disability Rating Index, where 0 points indicated no disability and 100 points indicated complete disability. The overall weighted mean score for patients treated with IMN was 30 points, ranging from 29.8 to 32.1, while for those treated with MIPPO, the score was 34 points, ranging from 33.8 to 39.2. Importantly, there was no significant difference between the two treatment methods, with a WMD of -0.12 and a 95% CI ranging from -0.3 to 0.09 (P = 0.25). Furthermore, no heterogeneity was observed in this analysis (I^2^ = 0%).

**Figure 23:**
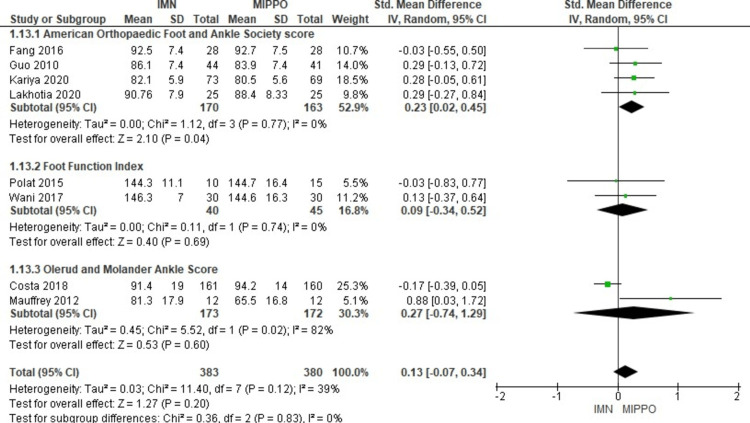
Forest plot for Functional ankle scores

In the evaluation of functional ankle scores at six to 12 months postoperatively, data from eight studies involving 763 patients were considered (Figure 23). These studies utilized various scoring systems, including the AOFAS score, Foot Function Index, and OMAS. Importantly, there was no significant difference in functional ankle scores between patients treated with IMN and those treated with MIPPO. The weighted standardized mean difference (WSMD) was 0.1, with a 95% CI ranging from -0.07 to 0.3 (P = 0.2). However, moderate heterogeneity was observed in this analysis (I^2^ = 39%). Subgroup analysis based on the type of functional score also revealed no statistically significant differences, with a test for subgroup differences yielding a P-value of 0.83.

Discussion

The purpose of this study was to investigate which minimally invasive treatment was more suitable for distal tibial fractures by comparing IMN and MIPO in 13 RCT studies. This review revealed that IMN has shorter time to union, time to full weight-bearing, and operation time, as well as a lower infection rate, whether superficial or deep infection. On the other hand, MIPPO was superior to IMN in reducing the malunion rate and anterior knee pain. No significant differences were found in non-union, radiation time, secondary operations, and general quality of life scores between the two methods. Functional outcomes assessed by FFI and OMAS were similar, but the AOFAS score favored MIPPO over IMN.

In this review, included studies utilized the term "extra-articular distal tibial fractures," but they referred to different regions, such as the distal tibial metaphysis, meta-diaphyseal junction, and adjacent diaphysis. These variations in criteria for defining distal tibial fractures led to different associations with AO/OTA classification types. Some studies focused solely on AO 42, while others concentrated on AO 43A, and some encompassed both AO 42 and AO 43A. To address these differences, we conducted subgroup analyses based on the AO/OTA classification and fracture type (closed or open), categorizing them into 42 closed, 42 closed or open, 43A closed, and mixed 42 and 43A groups according to the included studies. Notably, this review distinguishes itself from previous studies by including more RCTs with larger sample sizes and by performing subgroup analyses based on AO/OTA classification and fracture type.

Interpreting the results, it is clear that there are notable differences between the MIPPO technique and IMN.

Regarding infections, most studies divided it into superficial and deep infection. From a clinical point of view, deep infections are the most relevant of the two, as they frequently lead to re-intervention and/or prolonged duration of antibiotic treatment, while superficial infections are often resolved by oral antibiotics [24]. In this review, there was a higher risk of both superficial and deep infection among patients treated with MIPPO compared to IMN. In the MIPPO technique, 15% developed a superficial infection versus 7% after IMN, and 14% developed a deep infection versus 6.3% after IMN. Compared with the previous meta-analysis, the results are consistent with most of the previous studies, such as Liu et al. [25], Bleeker et al. [26], and Ekman et al. [27]. However, in a meta-analysis conducted by Kwok et al. [28], they did not find any significant difference in the incidence of infection in both the MIPPO and IMN methods.

Although the pooled data of all fracture types in this review revealed that IMN had a significantly lower incidence of infection than MIPPO, the subgroup analysis showed that patients whose fractures were classified as closed or open (AO 42 or 43A) seemed to be more inclined to have no statistical difference between both fixation methods.

The subgroup analysis focusing on closed fractures revealed an interesting finding. Among patients treated with MIPPO, those with fractures classified as AO 43A had a significantly higher incidence of infection compared to AO 42. This observation could be attributed to the slender soft tissue envelope on the medial aspect of the tibia, longer operation times, and the introduction of the plate in a MIPPO fashion on the medial side of the tibia, potentially compromising the insertion wounds.

Regarding malunion, most studies defined as varus/valgus deformity >50 in the coronal plane, anterior/posterior angulation >100 in the sagittal plane, a rotational deformity >100, and shortening >10 mm [21]. The pooled data pointed to a higher incidence of malunion in the IMN than the MIPPO. Malunion occurred in 14.7% of the patients after IMN and 8.8% after MIPPO fixation. Compared to the previous meta-analyses, some authors had the same finding as Liu et al. [25], Bleeker et al. [26], and Kwok et al. [28], while others found no difference as Wang et al. [29]. This controversy can be explained by the experience difference between surgeons in the included studies to achieve accurate fracture reduction and the variations in nail design and operative techniques.

This review revealed that the AO 42 fracture type had significantly higher rotational malalignment in IMN than MIPPO, but they did not find any significant difference in varus or valgus deformity and anterior/posterior angulation. Moreover, in AO 43A fracture type, IMN had significantly higher coronal plane deformity especially valgus deformity than MIPPO.

Kariya et al. [18] reported on malunion and the role of fibular fixation in improving rotational malalignment in the IMN group compared to conservatively managed fibular fracture in the AO 42 fracture type, and IMN had no significantly lower rotational malalignment than MIPPO.

In an effort to overcome the malunion complication, recent changes in IMN design and adjunctive techniques have been applied to maintain the reduction and alignment, such as angle-stable and multidirectional distal screws and block screws [30]. Blocking screws were used as a reduction tool to reduce the coronal deformity when applied in an anteroposterior direction and reduce the sagittal deformity when applied in the mediolateral direction [31]. In addition, the indirect reduction technique used in MIPPO may result in a similar rate of malunion to IMN [22,23].

In terms of the non-union rate, 11 studies reported detailed data about the incidence of non-union. Two of them reported no cases of non-union between the IMN and MIPPO techniques [21,22]. Non-union was defined as the lack of any healing within six months. The pooled data revealed no statistically significant difference in the risk of non-union between both treatment methods. The incidence of non-union occurred in 3.5% of patients in both treatment methods. The results are consistent with a previous meta-analysis as Wang et al. [29], Liu et al. [25], Bleeker et al. [26], and Ekman et al. [27]. Although the etiology of non-union is multifactorial, the surgical technique is well known to be one of the most important determinants of the union. As both surgical techniques are minimally invasive, they do not disrupt the fracture hematoma and impair the healing process.

Moreover, there was a trend toward non-union with fibula fixation. Kariya et al. [18] reported on the role of fibular fixation in non-union complications and found no difference in the non-union rate with or without fibula fixation, and there is no consensus on fibular fixation. As a result, in distal tibia fractures, fibula fixation is not recommended routinely. Fibula fixation should be reserved for spiral oblique and severely comminuted distal tibia fractures where the fibula can help maintain rotation and length.

In addition to union rate, the union time was reported in 10 studies. Union was defined as a dense callus bridging at least three of four cortices on biplanar radiographs. The pooled data showed that the IMN was significantly shorter compared to the MIPPO technique, and the mean time for union was 18 weeks in IMN compared to 20 weeks in MIPPO. This is consistent with previous meta-analyses, such as Wang et al. [29], Bleeker et al. [26], and Goh et al. [29]. Subgroup analysis showed that IMN had a significantly faster time to union in the AO 43A fracture type by 2.7 weeks' difference than MIPPO fixation with no significant difference in the AO 42 fracture type. Moreover, closed fractures, whether AO 42 or AO 34A, showed a significantly faster time to union than closed or open (AO 42 or AO 34A). The explanation may be that IMN provides relative stability that promotes secondary fracture healing. It is important to acknowledge that the assessment of time to union relies on radiographic evaluation using plain radiographs. This method can be challenging and is susceptible to significant interobserver variability [32].

Regarding anterior knee pain, it is well established that nailing tends to result in more anterior knee pain compared to plating, primarily because MIPPO does not involve any incision around the knee. In this study, anterior knee pain was observed in 28% of cases (66 out of 220 patients) and was the primary reason for implant removal [33,34]. It is worth noting that the etiology of anterior knee pain following intramedullary nailing is multifactorial, and proximal nail protrusion was not a significant contributing factor. However, nail removal can alleviate symptoms in many cases. Therefore, the relatively high incidence of anterior knee pain need not be the sole deciding factor when choosing between MIPPO fixation or IMN. Importantly, this issue applies to both nailing techniques, whether using an infra-patellar or supra-patellar approach [35,36].

Regarding operation and radiation time, IMN demonstrated a significantly shorter operation time compared to plating, with times of 74.1 minutes for IMN and 85.4 minutes for plating. Subgroup analysis indicated no significant difference in the AO 42 closed subgroup between the two treatment methods. However, there was a significant difference observed in the AO 42 closed or open and AO 43A subgroups. As for radiation time, data from four articles were analyzed, and the results of this meta-analysis revealed no significant difference between both treatment methods.

Concerning time to full weight-bearing, a comprehensive analysis was conducted based on data from seven articles. The results revealed a significantly faster time to full weight-bearing after IMN fixation, with a difference of approximately two weeks. However, it is important to acknowledge that assessing time to full weight-bearing is a subjective outcome ideally measured day to day. In the studies included in this meta-analysis, the ability to fully weight bear was assessed at fixed intervals due to study design limitations. Consequently, it is challenging to precisely gauge how this two-week difference translates into a clinical setting and how patients perceive this contrast in their recovery.

Regarding secondary operations, the pooled data revealed that both treatment methods have a high incidence of secondary operations with no significant difference between them. Secondary operations were required in 25% of the IMN method and 33% in the MIPPO fixation method. Furthermore, subgroup analysis showed no significant difference between different AO/OTA classification types of distal tibial fractures. Secondary operations included all reoperations during follow-up for treating infection, malunion, or non-union or for implant irritation symptoms in both methods.

Regarding the functional outcome, the functional assessment included various objective and subjective outcome scores such as the AOFAS score [11], OMAS [12], FFI [10], and DRI [9]. In the current study, there was no significant difference between the IMN and MIPPO groups in the quality-of-life score and functional scores, but MIPPO was superior to IMN in the AOFAS score. As the studies included in this meta-analysis used different functional outcome scores to assess the functional outcomes, the pooled scores of the AOFAS, FFI, and OMAS remained controversial.

Implications for Further Research

The existing literature provides ample evidence that both MIPPO fixation and IMN are viable options for treating distal tibia fractures. Future studies should place greater emphasis on determining the optimal treatment approach while considering the risk of infection, malunion, and anterior knee pain as crucial parameters. Currently, there are limited data on tailoring treatment on a case-by-case basis. Therefore, future research should focus on aligning surgical decision-making with individual factors, including comorbidities, physical condition, and social factors, to optimize patient outcomes.

In addition, when considering the fracture pattern and the soft-tissue status of each patient, it is essential to take into account adjuvant reduction techniques, advancements in implant design, and innovations in surgical approaches. Surgeons should be aware of the advantages and disadvantages of each technique in terms of alignment, stability, and soft-tissue biology. This understanding will aid in making informed decisions and selecting the most suitable technique for a specific fracture case.

Limitations of the Analysis

This analysis acknowledges several limitations that warrant consideration. First, a noteworthy proportion of the included RCTs featured relatively small patient cohorts, potentially resulting in underpowered results, particularly when evaluating functional outcomes. This limitation underscores the importance of interpreting functional outcome findings with caution.

Second, there was notable variability in the terminology used to describe infection rates, specifically with regard to "superficial and deep" infections. This variability across the majority of included studies introduces subjectivity and heterogeneity, posing a challenge in achieving standardized comparisons. The establishment of a consensus definition for fracture-related infection by Metsemakers et al. in 2018 [37] may pave the way for greater uniformity in future studies, enhancing the precision of infection rate assessments.

Third, the availability of data on radiation time was limited, with only four RCT studies contributing to this parameter. This scarcity of data may contribute to the observed high heterogeneity and render subgroup analysis less reliable for this specific outcome.

Fourth, the diverse use of functional outcome scores across studies prevented a pooled analysis, restricting the number of studies eligible for assessing functional outcomes. This diversity in scoring systems emphasizes the need for standardization in reporting functional outcomes to facilitate more robust comparisons in future analyses.

Finally, variations in surgical techniques and devices employed in each study introduce an additional layer of complexity. For example, the discretion allowed for fibula fixation by some studies may influence the risk of malunion; albeit potentially reducing the risk of malunion, it has also been associated with an elevated risk of delayed union and non-union [38,39]. These variations underscore the need for careful consideration of the specific methodologies employed in each study when interpreting treatment efficacy and complication rates.

## Conclusions

The findings of this study underscore the comparable therapeutic efficacy of both MIPPO and IMN in addressing distal tibial extra-articular fractures. While IMN presents distinct advantages, including a lower incidence of infection, diminished implant irritation, shorter operation times, and expedited weight-bearing and union, it is noteworthy that this method is associated with a higher prevalence of malunion and anterior knee pain.

The decision regarding the optimal implant choice necessitates a nuanced approach, taking into account individual patient characteristics. Patients at an elevated risk of infection, characterized by factors, such as advanced age, comorbidities, smoking history, or severe soft tissue injuries, may find greater benefit in opting for nail fixation. Conversely, for young, active, and healthy patients less prone to infections, desiring to mitigate the risk of knee pain and malunion, MIPPO fixation emerges as a favorable alternative. This individualized approach ensures that the chosen intervention aligns optimally with the unique needs and circumstances of each patient, thereby enhancing overall treatment outcomes.
